# The 21-gene recurrence score complements IBTR! Estimates in early-stage, hormone receptor-positive, HER2-normal, lymph node-negative breast cancer

**DOI:** 10.1186/s40064-015-0840-y

**Published:** 2015-01-30

**Authors:** Nikhil G Thaker, Karen E Hoffman, Michael C Stauder, Simona F Shaitelman, Eric A Strom, Welela Tereffe, Benjamin D Smith, George H Perkins, Lei Huo, Mark F Munsell, Lajos Pusztai, Thomas A Buchholz, Wendy A Woodward

**Affiliations:** Department of Radiation Oncology, Unit 1202, The University of Texas MD Anderson Cancer Center, 1515 Holcombe Boulevard, Houston, TX 77030 USA; Department of Pathology, The University of Texas MD Anderson Cancer Center, Houston, TX USA; Department of Biostatistics, The University of Texas MD Anderson Cancer Center, Houston, TX USA; Department of Medical Oncology, Yale Cancer Center, New Haven, CT USA

**Keywords:** Oncotype Dx, 21-gene recurrence score, Molecular profiling, Ipsilateral breast tumor recurrence, IBTR!, Local recurrence

## Abstract

Clinicians have traditionally used clinicopathological (CP) factors to determine locoregional recurrence (LR) risk of breast cancer and have generated the IBTR! nomogram to predict the risk of ipsilateral breast tumor recurrence (IBTR). The 21-gene recurrence score (RS) assay was recently correlated with LR in retrospective studies. The objective of this study was to examine the relationship between the RS and IBTR!. CP characteristics of 308 consecutive patients who underwent RS testing at our institution were examined. IBTR! was used to estimate the risk of 10-year IBTR. Descriptive statistics were used to compare the RS with the estimated IBTR!. Given a low event rate in this cohort, actual IBTR rates were not reported. Most patients had stage I/II (98%) and grade I/II (77%) disease. Median age was 54 years (range, 30–78). Median IBTR! without radiation therapy was 10% (mean, 12% [range, 4-43%]). RS was low (<18), intermediate (18–30), and high (>30) in 52% (n = 160), 40% (n = 123), and 8% (n = 25) patients. Overall, IBTR! did not correlate with RS (*P* = .77). We saw no correlation between RS and IBTR! in patients with less than (*P* = .32) or greater than (*P* = .48) a 10% risk of IBTR. Interestingly, Ki-67 expression correlated with both IBTR! (*P* = .019) and the RS (*P* = .002). Further study is warranted to determine if the RS can provide complementary biological information to CP factors in estimating the risk of LR. Prospective studies evaluating this association may potentially allow for individualized treatment decisions.

**Electronic supplementary material:**

The online version of this article (doi:10.1186/s40064-015-0840-y) contains supplementary material, which is available to authorized users.

## Introduction

Local treatment decisions for patients with breast cancer are traditionally based on conventional clinicopathological (CP) factors such as age, tumor size and grade, TNM stage, margin status, lymphovascular invasion (LVSI), chemotherapy use, and hormone therapy use. A web-based predictive nomogram IBTR! (IBTR Version 2 0 Breast Cancer Model) is commonly used to estimate a patient’s risk of ipsilateral breast tumor recurrence (IBTR) according to several well-established CP factors (Sanghani et al. 2007; Sanghani et al. 2010). However, breast cancer is actually a heterogeneous group of diseases, and definitive treatment options may vary by molecular subtype.

Physicians have incorporated several molecular profiling tools into breast cancer clinics (Paik et al. 2004; Goldstein et al. 2008; Tang et al. 2011). The most widely used gene expression profiling tool is the 21-gene Oncotype Dx Recurrence Score (RS) assay (OncotypeDX; GenomicHealth Inc., Redwood City, Calif.), which is used to estimate the risk of 10-year distant recurrence (DR) and predict the likelihood of benefit of adjuvant chemotherapy for hormone receptor (HR)-positive, node-negative, tamoxifen-treated breast cancer (Paik et al. 2004; Paik et al. 2006). Both the National Comprehensive Cancer Network and American Society of Clinical Oncology consider the 21-gene RS assay an option for evaluating early-stage, HR-positive, lymph node-negative breast cancer (Network 2013; Harris et al. 2007).

Despite rapid integration of these genetic tools to assess the need for systemic management of breast cancer, their use in estimating the risk of locoregional recurrence (LR), which includes IBTR, chest wall recurrence, and regional nodal recurrence, has remained underdeveloped. Recent studies suggest that molecular profiling tools designed to assess the risk of DR, such as the RS, also predict increased risk of LR (Haffty 2002; Voduc et al. 2010; Mamounas et al. 2010; Taghian et al. 2004; Wapnir et al. 2008; Kirk 2010; Cheng et al. 2006; Nuyten et al. 2006). However, detailed information about the overlap between molecular-based risk stratification and standard CP factors that are known causes of LR is unavailable. Elucidation of this relationship would be useful, because clinicians have traditionally utilized CP factors to predict the risk of IBTR, and molecular tools hold the future promise for predicting the risk of LR. The objective of the present study was to examine the relationship between the 21-gene RS and the estimated risk of IBTR based on CP features in breast cancer patients primarily at intermediate-risk for IBTR, for whom clinical uncertainty exists as to the need for therapeutic escalation versus de-escalation. We sought to identify clinical scenarios in which the RS may complement CP information when making individualized local management decisions.

## Materials and methods

### Study population

Patients diagnosed with breast cancer who underwent the 21-gene RS assay testing as part of routine care or in the context of the National Cancer Institute’s Trial Assigning IndividuaLized Options for Treatment (TAILORx) trial from December 2004 to November 2008 were included in this study. In the TAILORx trial, researchers are evaluating whether hormone therapy alone or with combination chemotherapy is better for women with node-negative, estrogen receptor (ER)-positive breast cancer having an RS of 11–25 (Hormone Therapy With or Without Combination Chemotherapy in Treating Women Who Have Undergone Surgery for Node-Negative Breast Cancer (The TAILORx Trial) 2013). Patients who were clinically node-negative but pathologically node-positive during this time period were also included in this cohort as part of routine care at this institution.

Demographic and CP data on the study patients were abstracted from a prospectively maintained clinical database according to an MD Anderson Institutional Review Board-approved protocol. All pathological information, including histological type, tumor grade, tumor size, nodal status, and tumor marker expression were reviewed by breast pathologists. Ki-67 expression was measured using immunohistochemistry as part of the routine pathological evaluation and was reported as the percentage of Ki-67 positive nuclei in invasive neoplastic cells. Ki-67 expression information was available for 116 of 308 patients.

### The 21-gene RS

Tumor samples obtained from the study patients were submitted for the 21-gene RS assay (OncotypeDX; GenomicHealth Inc., Redwood City, Calif.). This profiling tool measures the expression of 16 cancer-related and 5 control genes in paraffin-embedded breast tumor samples using a reverse transcriptase-polymerase chain reaction assay as described previously (Paik et al. 2004). Based on the level of expression of each gene, a continuous variable known as the RS is calculated, ranging from 0 to 100. This score can be used to estimate the risk of 10-year DR and predict the likelihood of adjuvant chemotherapy benefit in patients with node-negative, HR-positive, tamoxifen-treated breast cancer (Paik et al. 2004; Paik et al. 2006).

### IBTR! nomogram

The IBTR! nomogram (version 2.0; Breast Cancer Model) is a web-based predictive tool that uses literature-derived relative risk ratios for seven CP tumor factors (age, tumor size and grade, margin status, LVSI, and chemotherapy and hormone therapy use) to predict the 10-year risk of IBTR with and without radiation therapy (RT) (Sanghani et al. 2010). The 10-year estimated risk of IBTR was calculated and recorded for each patient using IBTR! (IBTR Version 2 0 Breast Cancer Model; Sanghani et al. 2010). IBTR! without RT was used in this study (Sanghani et al. 2010).

### Statistical analysis

Descriptive statistics were used to compare the RS with the estimated 10-year risk of IBTR!. Analysis of variance with Tukey’s adjustment for multiple comparisons was used to compare scores among various factors. Correlations among RS, age, and Ki-67 expression were also evaluated. Spearman’s rank correlation coefficient was used to estimate the strength of association between RS and IBTR!, RS and Ki-67 expression, IBTR! and Ki-67 expression, and IBTR! and age at diagnosis. Ki-67 was compared as a categorical variable and as a continuous variable. Stratification according to type of local treatment was also include for the IBTR! and RS comparisons. All significance tests were two-tailed, with *P* values less than .05 considered significant. Analyses were performed using the SAS software program (version 9.3 for Windows; SAS Institute Inc., Cary, N.C.). Actuarial LR, IBTR!, and survival results were not reported because of a short median follow-up duration and low event rate.

## Results

### Patient and tumor characteristics

We identified 308 consecutive patients who underwent the RS assay (Table 1). Their median age was 54 years (range, 30–78 years). Ninety-nine percent of patients (N = 305) had stage I/II disease, and 77% (N = 238) had grade I/II tumors. Sixty-six percent (N = 203) underwent breast-conserving surgery (BCS; 6% [N = 19] alone and 60% [N = 184] with RT), and 34% (N = 105) underwent mastectomy (31% [N = 96] alone and 3% [N = 9] with RT). We classified 52% (N = 160), 40% (N = 123), and 8% (N = 25) of the patients into the low-risk (<18), intermediate-risk (18–30), and high-risk (>30) categories as defined by the assay. Using the TAILORx risk groups, we classified 69% (N = 212) of the patients into the intermediate-risk group (RS of 11–25).

**Table 1:** **Patient demographics and characteristics**

		**No. of patients (%)**			
**Variable**	**All patients, N = 308**	**Low RS (<18), N = 160**	**Intermediate RS (18–30), N = 123**	**High RS (>30), N = 25**	***P*** **-value**
Median age at diagnosis, y (range)	54 (30–78)	53 (31–78)	55 (30–73)	59 (40–75)	NS
Frequency by race					NS
Asian/Pacific Islander	10 (3)	7 (4)	2 (2)	1 (4)	
Black	16 (5)	7 (4)	7 (6)	2 (8)	
Native American	1 (0.3)	1 (1)	0 (0)	0 (0)	
Other	2 (1)	0 (0)	1 (1)	1 (4)	
Spanish/Hispanic	42 (14)	24 (15)	14 (11)	4 (16)	
White	237 (77)	121 (76)	99 (81)	17 (68)	
Type of surgery					NS
BCT	203 (66)	100 (63)	85 (69)	18 (72)	
Mastectomy	105 (34)	60 (38)	38 (31)	7 (28)	
Histology					.0225
Invasive ductal	214 (70)	100 (63)	91 (74)	23 (92)	
Invasive lobular	37 (12)	24 (15)	13 (11)	0 (0)	
Other	57 (19)	36 (23)	19 (15)	2 (8)	
Pathological stage					NS
0	3 (1)	3 (2)	0 (0)	0 (0)	
I	243 (79)	127 (79)	97 (79)	19 (76)	
IIA	52 (17)	25 (16)	21 (17)	6 (24)	
IIB	7 (2)	3 (2)	4 (3)	0 (0)	
IIIA	1 (0.3)	1 (1)	0 (0)	0 (0)	
IIIB	1 (0.3)	1 (1)	0 (0)	0 (0)	
No. of positive lymph nodes					NS
0	286 (93)	152 (95)	110 (89)	24 (96)	
1	15 (5)	4 (3)	10 (8)	1 (4)	
2	2 (1)	0 (0)	2 (2)	0 (0)	
3	1 (0.3)	1 (1)	0 (0)	0 (0)	
4	1 (0.3)	1 (1)	0 (0)	0 (0)	
Tumor grade					<.0001
I	45 (15)	31 (19)	14 (11)	0 (0)	
II	193 (63)	107 (67)	80 (65)	6 (24)	
III	70 (23)	22 (14)	29 (24)	19 (76)	
PR status					<.0001
Positive	272 (88)	145 (91)	112 (91)	15 (60)	
Negative	31 (10)	10 (6)	11 (89)	10 (40)	
Unknown	5 (2)	5 (3)	0 (0)	0 (0)	
Ki-67 expression					.0024
High (>20%)	27 (9)	9 (6)	11 (9)	7 (28)	
Intermediate (10-20%)	41 (13)	23 (14)	16 (13)	2 (8)	
Low (<10%)	48 (16)	29 (18)	19 (16)	0 (0)	
Not measured	195 (63)	99 (62)	77 (63)	16 (64)	
Vascular invasion					.0162
Positive	41 (13)	16 (10)	17 (14)	8 (32)	
Negative	267 (87)	144 (90)	106 (86)	17 (68)	
Tumor size					NS
<2 cm	230 (75)	119 (75)	93 (76)	18 (72)	
≥2 cm	73 (24)	38 (24)	28 (23)	7 (28)	
Chemotherapy use					<.0001
Yes	81 (26)	15 (9)	47 (38)	19 (76)	
No	227 (74)	145 (91)	76 (62)	6 (24)	
Hormone therapy use					.0220
Yes	264 (86)	145 (91)	100 (81)	19 (76)	
No	44 (15)	15 (9)	23 (19)	6 (24)	

*Abbreviations: BCT* breast conserving therapy, *NS* not significant, *PR* progesterone receptor.

### Correlation of CP factors with 21-gene RS

High (>20%) Ki-67 expression (Delpech et al. 2012; Kilickap et al. 2014), high tumor grade, LVSI, and chemotherapy use was seen in a greater proportion of the high RS group than in the low and intermediate RS groups. However, we saw no differences in pathological stage, type of surgery (BCS versus mastectomy), number of positive lymph nodes, or tumor size among the risk groups (Table 1). When we stratified the CP factors by type of surgery (BCS versus mastectomy), we observed no significant differences according to RS risk group, tumor grade, LVSI, or chemotherapy use. However, patients who underwent mastectomy tended to have larger primary tumors (*P* = .0069) and more hormone therapy use (*P* = .0406) than did patients who underwent BCS.

### Correlation of IBTR! score and RS

**Figure 1:**
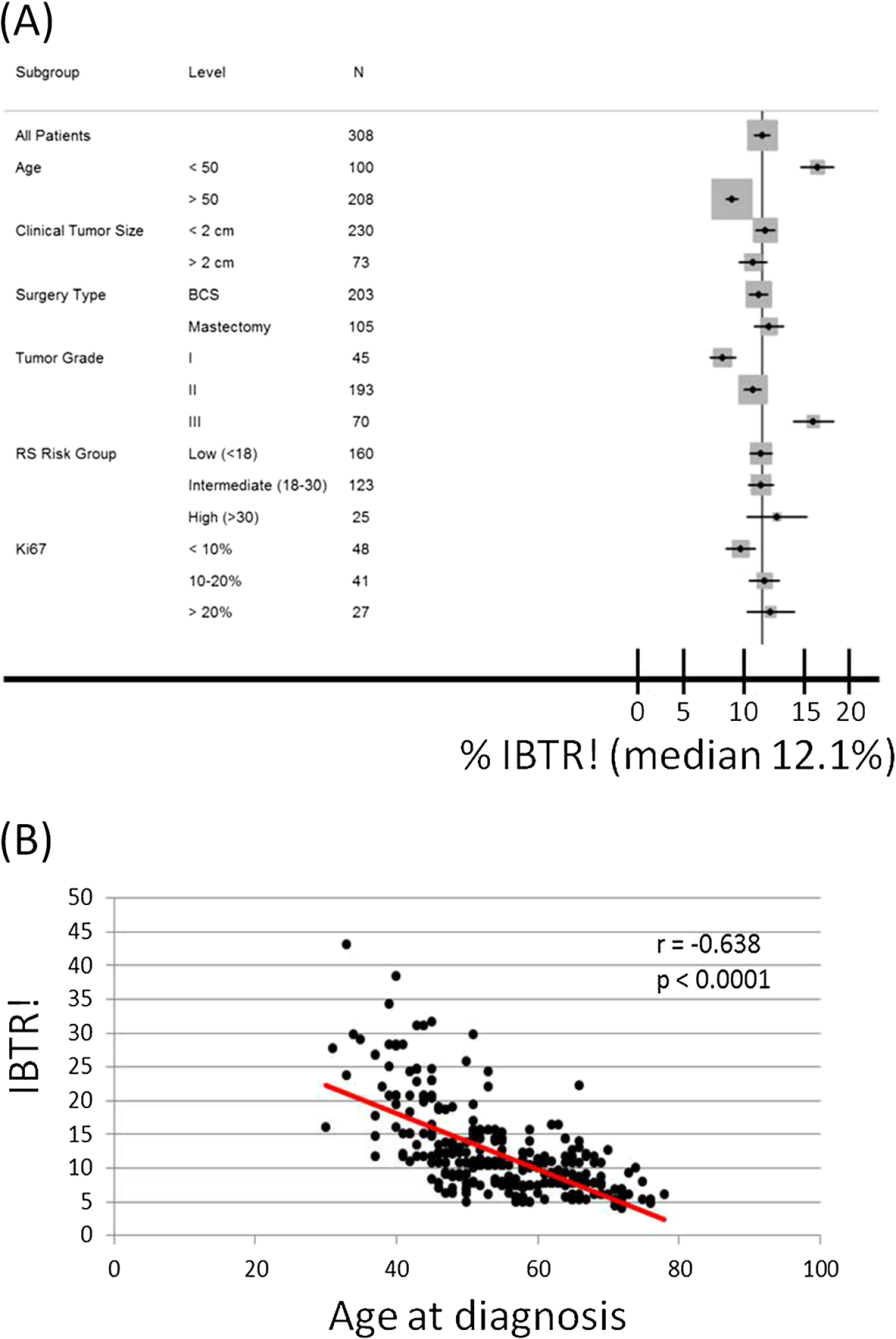
**IBTR! score according to patient and tumor characteristics. (A)** IBTR! stratified according to age, tumor size, surgery type, tumor grade, RS risk group, and Ki-67 expression. **(B)** IBTR! according to age at diagnosis.

**Figure 2:**
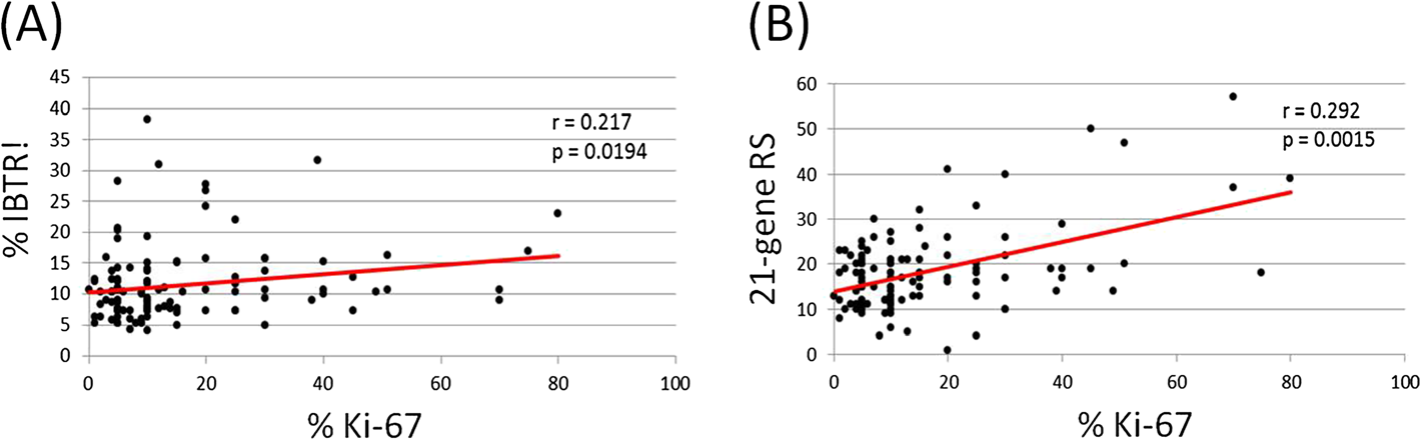
**Correlation of Ki-67 expression with (A) IBTR! score and (B) RS.**

**Figure 3:**
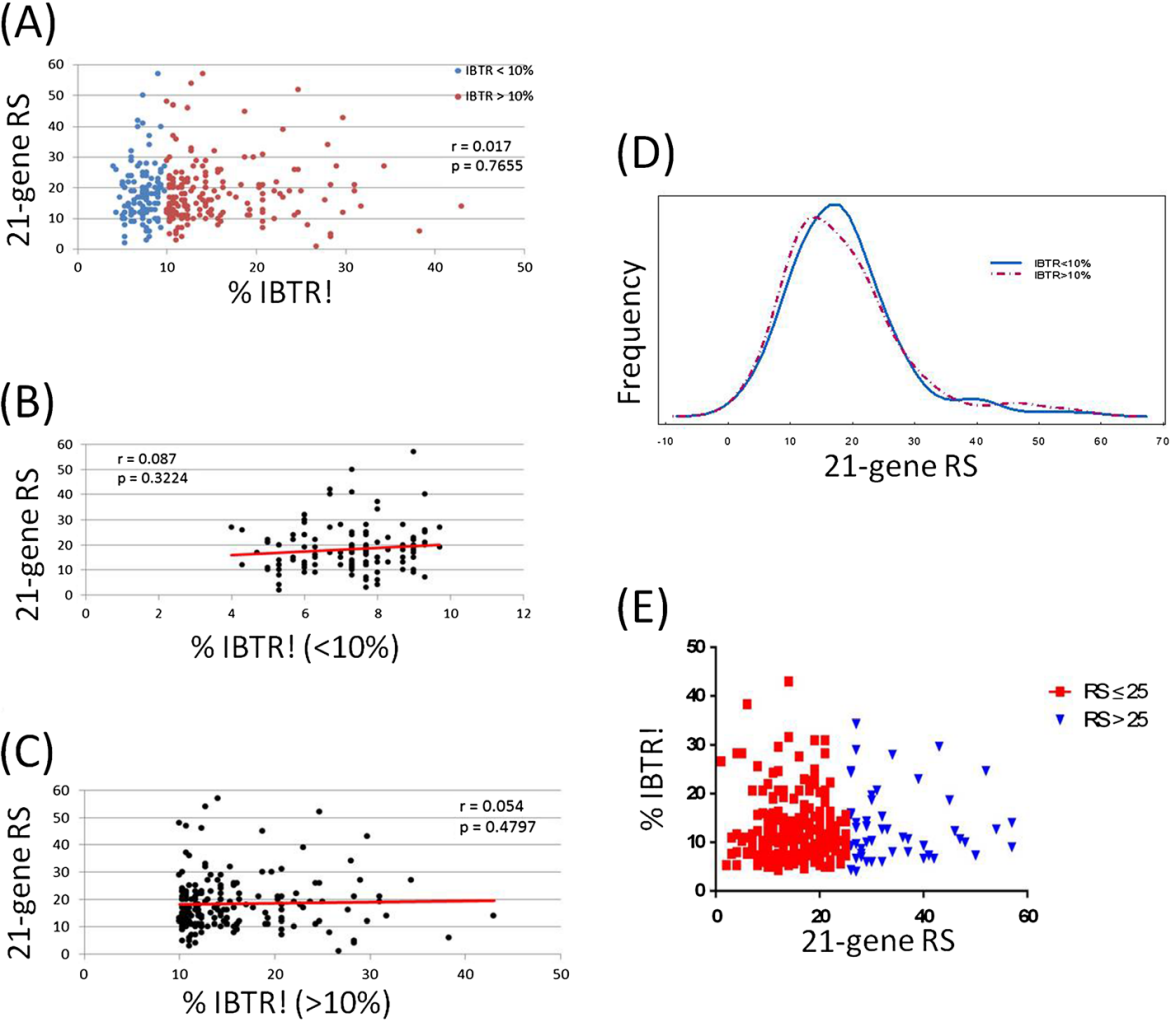
**Comparisons of the IBTR! score according to the 21-gene RS. (A)** The 21-gene RS versus estimated IBTR! score according to CP factors. **(B and C)** The 21-gene RS versus IBTR! when the IBTR! was **(B)** less than 10% and **(C)** greater than 10%. **(D)** The frequency distribution of IBTR! score according to 21-gene RS. **(E)** Scatter plot of the large variations in IBTR! in the TAILORx risk groups.

The median IBTR! score in all 308 patients was 10% (mean, 12% [range, 4-43%]). As expected, young age (*P* < .001) and high tumor grade associated most strongly with higher IBTR! scores (Figure 1) (*P* < .001). Interestingly, Ki-67 expression correlated with both IBTR! and RS (Figure 2A, B). However, IBTR! score did not correlate with RS (*P* = .7655, r = .017) (Figure 3A). Among patients with less than a 10% risk of IBTR (n = 132), 50% had low RSs, whereas the remaining 50% had intermediate or high RSs (Figure 3B). Likewise, we did not observe significant correlation of IBTR! score with RS at higher IBTR! scores (Figure 3C). Additionally, there was no significant difference in the distribution of RS when IBTR was low (<10%) or high (>10%) (Figure 3D). Stratification of IBTR! scores according to TAILORx risk groups (RS < =25 and RS > 25) further emphasized the wide variation in IBTR! score with either a high or low to intermediate RS and no correlation (Figure 3E).

**Figure 4:**
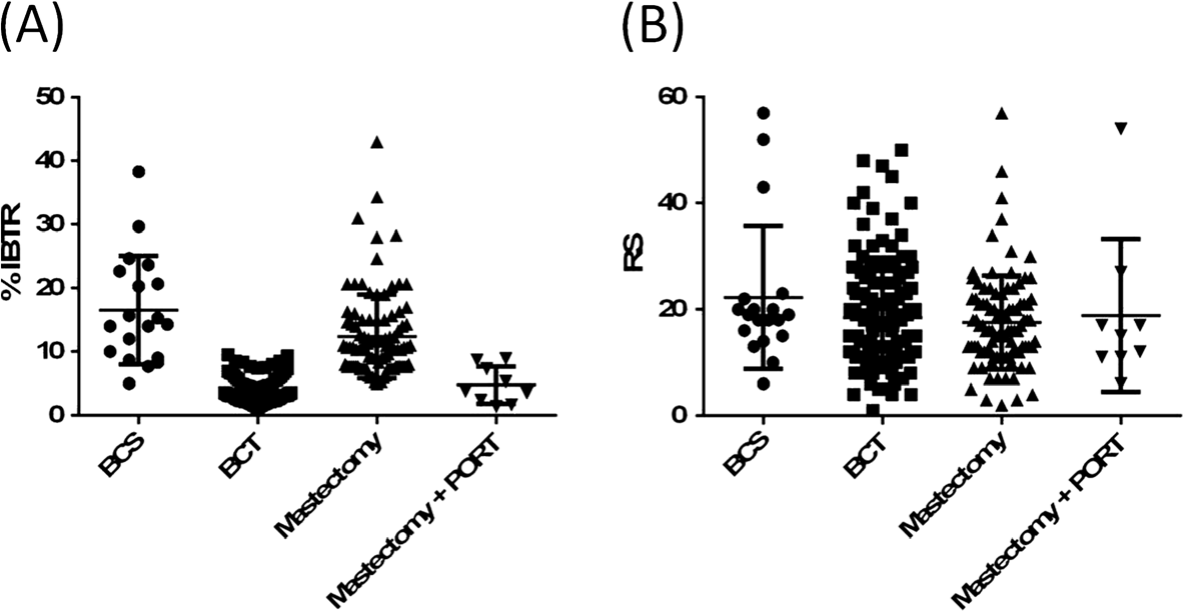
**IBTR! and the 21-gene RS according to actual treatments received. (A)** IBTR! score according to actual treatment (i.e., adjuvant RT). **(B)** RS according to actual treatment (i.e., adjuvant RT). The distribution in BCS and mastectomy subgroups are wide in both the IBTR! and RS stratifications. However, the deviation is less in the groups that have undergone RT. BCS, breast conservation surgery; BCT, breast conservation therapy; PORT, post-operative radiation therapy.

We then estimated the IBTR! scores for each of the four treatment groups, based upon the actual treatments received (BCS, breast conserving therapy [BCT], Mastectomy, and Mastectomy and postoperative radiation therapy [PORT]). Previous comparisons utilized the IBTR! without RT for all patients to provide a more homogeneous IBTR! score estimate. These comparisons once again confirmed that patients at high or low risk for IBTR! based on CP factors and treatment (Figure 4A) still had large variations in RS (Figure 4B), emphasizing the need to evaluate molecular profiles in homogeneously treated populations of breast cancer patients. The distribution in BCS and mastectomy subgroups are wide in both the IBTR! and RS stratifications. However, the deviation is less in the groups that have undergone RT.

## Discussion

Physicians have traditionally based LR treatment decisions for patients with early-stage breast cancer on CP characteristics such as tumor size and grade, margin status, pathological nodal status, and LVSI. However, breast cancer represents a heterogeneous group of diseases, and additional biological information about tumor gene expression may assist in local therapy decisions. Despite major progress in identifying genomic profiles associated with risk of DR and benefit of chemotherapy, only recently have studies demonstrated that the 21-gene RS may help estimate the risk of LR (Voduc et al. 2010; Mamounas et al. 2010). To date, though, researchers have yet to explicitly determine how many patients with seemingly innocuous breast cancer in terms of LR estimates may have high RSs or *vice versa*. The present study demonstrated minimal redundancy between CP risk factors and RS, suggesting that the 21-gene RS adds complementary biological information to the CP factors traditionally used to assess risk of IBTR.

Several promising studies recently examined the relationship between the RS and LR (Table 2). Mamounas *et al.* (Mamounas et al. 2010) initially examined the correlation between the 21-gene RS and LR risk in patients with node-negative, ER-positive breast cancer treated with breast conservation therapy (BCT) or mastectomy without RT. The authors concluded that RS was highly associated with risk of LR in patients with tamoxifen-treated, placebo-treated, or chemotherapy- plus tamoxifen-treated disease. RS, age, and initial treatment type were independent predictors of LR. However, the relationship was more complex in patients who received RT, suggesting that the effectiveness of RT increases as the RS increases. Solin *et al.* reported more conflicting results (Solin et al. 2012). They demonstrated that neither biological subtype nor 21-gene RS were associated with LR, although RS was a significant predictor as a continuous variable in patients with HR-positive tumors. The authors concluded that neither biological subtype nor 21-gene RS should preclude BCT with RT.

**Table 2:** **Summary of studies assessing the relationship of the RS and LR in breast cancer**

**Study**	**Molecular profiling tool**	**Population**	**Objective**	**Number of patients in the study**	**Outcomes and conclusions**
Thaker *et al.* (present study)	21-gene RS assay	Early-stage, HR-positive, lymph node-negative	Correlation between 21-gene RS and IBTR! nomogram	308	No correlation between 21-gene RS and IBTR! estimates; RS may complement traditional CP factors when assessing risk of IBTR in intermediate-risk patients
Mamounas *et al.*	21-gene RS assay	Tamoxifen-treated, node-negative, ER-positive disease treated with placebo, tamoxifen, or chemotherapy plus tamoxifen from two NSABP trials (B-14 and B-20)	Association between RS and risk of LR	895 tamoxifen-treated, 355 placebo-treated, 424 chemotherapy plus tamoxifen-treated	Significant association between RS and risk for LR; LR was significantly associated with RS risk group in tamoxifen-treated, placebo-treated, and chemotherapy- plus tamoxifen-treated patients; RS was an independent significant predictor of LR along with age and type of initial treatment
Solin *et al.*	21-gene RS assay	Operable breast adenocarcinoma with either 1–3 axillary lymph nodes involved or negative axillary lymph nodes with a primary tumor size >1 cm; patients with HR-positive tumors received adjuvant hormonal therapy; patients received AC versus AT chemotherapy as per ECOG E2197	Evaluation of the significance of the 21-gene RS and biological subtype relative to LR after BCT	388	Neither biological subtype nor 21-gene RS was associated with LR; for HR-positive tumors, the 21-gene RS evaluated as a continuous variable was significant for LR; neither biological subtype nor 21-gene RS should preclude BCT with RT
Mamounas *et al.*	21-gene RS assay	ER-positive, tamoxifen-treated, node-positive disease treated with adjuvant chemotherapy with AC versus AC-T in the NSABP B-28 trial	Association between RS and risk of LR	1065	RS was significantly associated with LR after lumpectomy and breast RT and after mastectomy (no RT) as well as in patients with ≥4 positive nodes (with a nonsignificant trend in patients with 1–3 positive nodes); in MVA, RS, nodal status, and tumor size were all independent predictors of LR
Solin *et al.*	DCIS RS assay	DCIS treated with surgical excision without RT in the ECOG E5194 study	Association of DCIS score (modified RS assay with 7 cancer-related genes and 5 reference genes) with risk of IBE	327	Continuous DCIS score was significantly associated with the risk of an IBE and invasive IBE; this score complements traditional CP factors

*Abbreviations: NSABP* National Surgical Adjuvant Breast and Bowel Project, *AC* doxorubicin plus cyclophosphamide, *AT* doxorubicin plus docetaxel, *ECOG* Eastern Cooperative Oncology Group, *AC-T* AC followed by paclitaxel, *MVA* multivariate analyis, *IBE* ipsilateral breast event (defined as local recurrence of DCIS or invasive carcinoma), *LR* locoregional recurrence.

More recently, several groups examined the prognostic and predictive ability of the 21-gene RS assay in other subgroups of breast cancer patients. For example, Mamounas *et al.* (Mamounas et al. 2013) reported a significant association between RS and LR after BCT or mastectomy in patients with at least four positive nodes and a nonsignificant trend in patients with one to three positive nodes. Solin *et al.* (Solin et al. 2013) modified the 21-gene RS assay to create a ductal carcinoma *in situ* (DCIS) RS, incorporating expression data on seven cancer-related genes and five reference genes. They found that the continuous DCIS RS was significantly associated with the risk of an ipsilateral breast event after BCS without RT. Nevertheless, the possibility that these tools are useful to identify patients not typically offered RT who are at higher risk than their CP factors estimate or to identify patients typically offered RT who may be safely observed is appealing. Prospective trials will be needed to confirm how RS segregates from clusters of standard CP factors.

While researchers have examined the correlations among CP factors and RSs, they have yet to report data assessing the range of scores within each subgroup that would typically be considered indicative of low or high risk of LR. From a chemotherapy perspective, 40% of the patients in our cohort had intermediate RSs ranging from 18 to 30; this group expanded to 69% with the use of TAILORx risk grouping, reflecting the high proportion of intermediate-risk clinical scenarios. Given the overlap between IBTR risk estimates based on CP factors and RS, our cohort also represents a clinically ambiguous group from an LR standpoint. Although IBTR! score did not correlate with RS in our study, the large variations in RS and IBTR! score suggest that these factors provide different information regarding the risk of IBTR. Interestingly, Ki-67 expression correlated with both RS and IBTR! score. Although Ki-67 expression is part of the RS calculation, it is not part of the IBTR! score calculation. This implies that cellular proliferation may represent a genetic driver of LR.

The present study demonstrates that breast cancer patients who are typically offered the 21-gene RS testing are clinically, pathologically, and perhaps molecularly heterogeneous. We included patients given treatment with various modalities, including BCS, BCT, mastectomy alone, and mastectomy with PORT. As further research is conducted, evaluating the risk of LR in homogeneously treated populations will be important, as the risk factors driving relapse after BCS or mastectomy may be different from those after BCT or PMRT. Neoadjuvant systemic therapy is yet another consideration when evaluating the molecular drivers of LR. Additionally, although researchers have suggested that LR and DR are linked, CP factors and molecular profiles associated with LR may differ from those that predict DR. For instance, Solin *et al.* (Solin et al. 2013) modified the constituent gene profile for the DCIS RS to better reflect known biological differences between invasive and *in situ* disease. More comprehensive genetic analyses may define additional or alternative genes that better predict LR than does the 21-gene RS assay used in this study.

Although exploratory in nature, our study has several limitations, including those inherent to retrospective studies. IBTR risk estimation using IBTR! is intended for the breast-conserved population, but we applied this analysis to patients who underwent either breast conserving surgery (BCS) or mastectomy. However, our objective was to model the risk for IBTR based on CP factors rather than the actual treatment received. The overall non-significant differences between the two treatment cohorts also suggests that we could indeed isolate and compare IBTR! based upon the CP factors alone. Furthermore, researchers have suggested that IBTR! overestimates the risk of IBTR in high-risk patients. We also could not account for factors other than those used in the IBTR! nomogram, as we did not examine actuarial event rate data. Importantly, IBTR! does not incorporate hormone receptor or HER2 status, and recent studies have suggested that receptor status can influence IBTR (Arvold et al. 2011).

Additionally, the IBTR! nomogram reports a point estimate of the risk of IBTR at 10-years, so continuous risk estimates before and after 10-years were not evaluable. Furthermore, our cohort comprised an overall favorable group of patients with node-negative disease. Consistent with previously published studies, (Paik et al. 2004; Mamounas et al. 2010; Solin et al. 2012; Mamounas et al. 2013) the actuarial event rates in such patients are low, requiring large patient cohorts with long follow-up durations for examination of actual outcomes. According to these data, we hypothesize that RSs may vary similarly in patients with node-positive breast cancer, for whom the decision to add a supraclavicular field to BCT or give postmastectomy radiation therapy (PMRT) may be most influenced by the addition of an independent factor that correlates with LR. However, our cohort did not have node-positive disease, and this relationship remains to be explicitly shown.

Despite these drawbacks, molecular profiling for LR continues to rapidly evolve. RS groups and their risks of IBTR are largely heterogeneous, and discordant RS values may provide information beyond that provided by standard CP factors. Indeed, integrating the RS with traditional CP parameters, treatment type, and patient-related factors may improve estimation of the risk of LR over use of any of these factors alone. Goldstein *et al.* and Tang *et al.* reported that combining the RS with traditional CP factors can improve prognosis for DR, (Goldstein et al. 2008; Tang et al. 2011) and such a clinical integrator may be an important new tool for estimating LR risk. The PAM-50 assay (Nanostring Prosigna assay) integrates clinical and molecular prognostic models to estimate the risk of later recurrence in postmenopasual, HR-positive breast cancer patients and is now commercially available. Future research efforts will require prospective assessment of these factors in large data sets to confirm, validate, and even integrate molecular signatures with CP factors in intermediate-risk patients. These data will help establish the number of patients with intermediate- and high-RS in low-risk subgroups required to screen in clinical trials, ultimately leading to personalization of treatment and improved outcomes.
